# Four Linked Genes Participate in Controlling Sporulation Efficiency in Budding Yeast

**DOI:** 10.1371/journal.pgen.0020195

**Published:** 2006-11-17

**Authors:** Giora Ben-Ari, Drora Zenvirth, Amir Sherman, Lior David, Michael Klutstein, Uri Lavi, Jossi Hillel, Giora Simchen

**Affiliations:** 1 Institute of Plant Sciences and Genetics, Faculty of Agriculture, Hebrew University of Jerusalem, Rehovot, Israel; 2 Samuel Lunenfeld Research Institute, Mount Sinai Hospital, Toronto, Ontario, Canada; 3 Department of Genetics, Hebrew University of Jerusalem, Jerusalem, Israel; 4 Agricultural Research Organization, Beit Dagan, Israel; 5 Stanford Genome Technology Center, Palo Alto, California, United States of America; 6 Department of Biochemistry, Stanford University School of Medicine, Stanford, California, United States of America; Princeton University, United States of America

## Abstract

Quantitative traits are conditioned by several genetic determinants. Since such genes influence many important complex traits in various organisms, the identification of quantitative trait loci (QTLs) is of major interest, but still encounters serious difficulties. We detected four linked genes within one QTL, which participate in controlling sporulation efficiency in *Saccharomyces cerevisiae.* Following the identification of single nucleotide polymorphisms by comparing the sequences of 145 genes between the parental strains SK1 and S288c, we analyzed the segregating progeny of the cross between them. Through reciprocal hemizygosity analysis, four genes, *RAS2, PMS1, SWS2,* and *FKH2,* located in a region of 60 kilobases on Chromosome 14, were found to be associated with sporulation efficiency. Three of the four “high” sporulation alleles are derived from the “low” sporulating strain. Two of these sporulation-related genes were verified through allele replacements. For *RAS2,* the causative variation was suggested to be a single nucleotide difference in the upstream region of the gene. This quantitative trait nucleotide accounts for sporulation variability among a set of ten closely related winery yeast strains. Our results provide a detailed view of genetic complexity in one “QTL region” that controls a quantitative trait and reports a single nucleotide polymorphism-trait association in wild strains. Moreover, these findings have implications on QTL identification in higher eukaryotes.

## Synopsis

Genes controlling many medically and agriculturally important complex traits in various organisms and their organization as quantitative trait loci (QTLs) are of major interest. To identify QTLs responsible for such a quantitative trait, the authors employed a two-step strategy: First, single-nucleotide markers (called SNPs) distributed throughout the genome were screened for prevalence among progeny with extreme characteristics, thus identifying three candidate genomic regions. Next, in one of these regions, manipulation of individual genes revealed four tightly linked genes that affected the trait, sporulation efficiency. A fifth gene that affects sporulation was recently and independently identified in the same region. This 60-kilobase region has a complex and interesting architecture: One strain, which sporulates efficiently, has sporulation-promoting alleles (alternative forms) at two major genes and inhibiting alleles at the three less important ones, whereas another strain, with inefficient sporulation, has the opposite alleles at the five genes. Moreover, one causative SNP for this trait, in the promoter region of the gene *RAS2,* explains sporulation differences among a set of ten winery yeast strains. These results provide a detailed view of genetic complexity in one “QTL region” and an SNP-trait association example among wild strains.

## Introduction

Analyses of quantitative trait loci (QTLs) have been carried out in various model organisms such as plants [1], rodents [2], *Drosophila* [3], and yeast [4], but the molecular and physiological basis for QTL differences is only partially understood. The genes affecting a quantitative trait may act additively, or interact non-additively with each other and with environmental factors. Amino acid changes [5,6], as well as changes in expression level, may result in functional variation [7–9]. Screening of candidate genes may be either based on specific genes in mapped intervals or through a genome-wide scan [10]. Several successful examples of candidate gene approaches for identification of QTLs have been reported recently [11–13].

In most cases, quantitative traits, like body weight or height, are characterized by one phenotypic value for each individual. Assessment of sporulation efficiency in the budding yeast Saccharomyces cerevisiae is based on evaluating the number of cells (within the same clonal genotype) that undergo the sporulation process. It is not clear what the factors are causing one cell to complete the sporulation process, while other, genetically identical cells of the same colony are arrested and do not complete the process. Furthermore, it is not always clear at which stage the cells are arrested. However, multiple measurements of sporulation efficiency on cell populations of the same genotype result in similar values. Therefore, the assumption is that in addition to environmental effects, sporulation efficiency is controlled by genetic factors and could serve as a model for analysis of quantitative traits, as has recently been demonstrated [14].

Analysis of pools made of equal amounts of DNA from several individuals saves on time and reduces the cost of large-scale genotyping projects. Determination of allele frequencies of single nucleotide polymorphisms (SNPs) in DNA pools, which has previously been assessed in several studies [15–22] was applied in the current study as well.

The aim of this study was to identify genes that control the differences in sporulation efficiency between the yeast strains SK1 (high sporulation efficiency) and S288c (low sporulation efficiency). Our approach to detect QTLs in S. cerevisiae was based on identification of genomic regions with significant differences in allele (SNP) frequencies between DNA pools of phenotypically similar segregants, followed by genetic manipulation of candidate genes therein. We thus identified four genes that are involved in the sporulation process and account for the differences in sporulation efficiency between the two laboratory strains. Moreover, we found that the QTL region contains alleles of opposing effects. We also identified one causative SNP in the promoter of the gene *RAS2,* which could explain sporulation variability among a set of ten closely related winery yeast strains.

## Results

Through sequencing of 81,480 base pairs (bp) in 145 genes from the SK1 genome, we discovered 554 SNPs that distinguish between SK1 and S288c. Of these genes, which were distributed throughout the genome (except for the smallest Chromosome 1), 132 had at least one SNP, and 46 had non-synonymous SNPs. The genes sequenced were known to be related to sporulation [23,24] (Table S1). For the purpose of the initial scan for polymorphisms, however, any collection of random SNPs distributed over the genome would have served us equally well.

**Figure 1:**
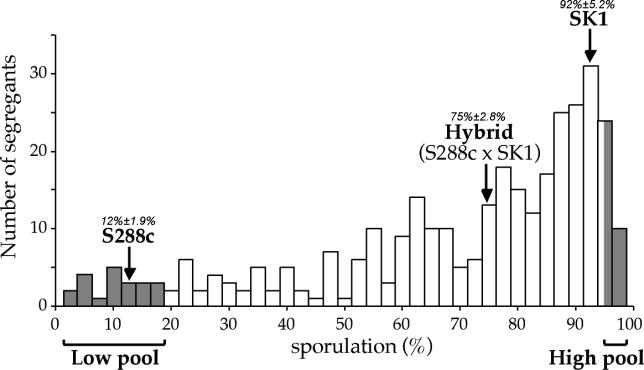
Distribution of Sporulation Efficiencies of Diploid Segregants Obtained from the Crosses S288c × SK1 Generation of diploid segregants is described in Materials and Methods. Sporulation efficiencies and standard errors of parents and hybrids are in italics. Assessment of sporulation was carried out after 7 d on solid sporulation medium. Equal amounts of DNA from 21 segregants at each of the two “tails” were pooled to generate the “high” and “low” DNA pools (gray bars).

Segregating progeny of the cross between the strains SK1 (sporulation efficiency 92% ± 5.2%) and S288c (sporulation efficiency 12% ± 1.9%) were used to identify QTLs that are responsible for differences in sporulation efficiency between these two strains. The 326 diploid segregants generated through meiosis of the hybrids S288c × SK1 (sporulation efficiency 75% ± 2.8%) varied between 1% and 97.5% in sporulation efficiency (Figure 1), as determined after 7 d on sporulation plates.

**Figure 2:**
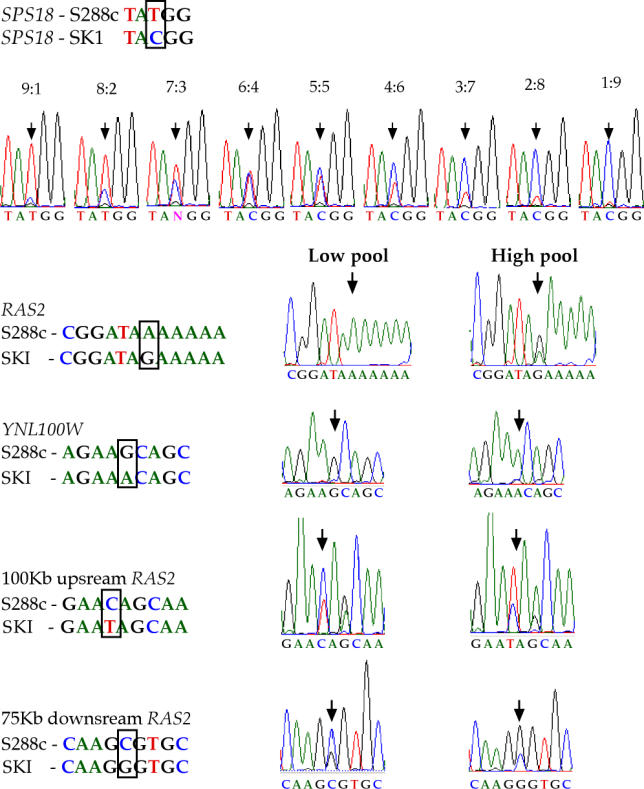
Sequences of DNA Pools Shown are short sequences with known SNPs. The upper part shows reconstruction of mixtures of DNA of the strains SK1 and S288c, testing the ability to evaluate reliable allele frequencies of SNPs in DNA pools. DNA of strains SK1 and S288c were pooled at various ratios and a short genomic region containing a known SNP in the gene *SPS18* was sequenced. The two alleles could clearly be distinguished even in pools with allele ratios of 8:2 and 9:1. The signal height of the DNA pool sequence was found to be a very good estimator for the allele frequency (correlation coefficient *r* = 0.99, *p* < 0.0001). The figure contains sequences of the “high” and “low” DNA pools from the genes *RAS2* and *YNL100W* and from polymorphic DNA segments flanking the candidate region on Chromosome 14. In each sequence, the SNP position is labeled by a black box or arrow (the SNP in the promoter of *RAS2* is in position −52).

DNA pools of 21 segregants from each “tail” of the sporulation efficiency distribution were used to evaluate the frequency of SNP alleles (“high” and “low” pools in Figure 2).

To verify the reliability of sequencing DNA pools, a preliminary reconstruction experiment was performed as follows: DNA of the strains SK1 and S288c were pooled at various ratios and a short genomic region containing a known SNP in the gene *SPS18* was sequenced. The two alleles could clearly be distinguished in pools with allele ratios of 8:2 and 9:1 (Figure 2). The height of the sequencing peak was found to be a good estimator for the allele frequency (correlation coefficient *r* = 0.99, *p* < 0.0001).

For each of the 132 genes that contained SNPs distinguishing between SK1 and S288c, one polymorphic DNA fragment was sequenced in the two DNA pools, and allele frequencies were compared between them. With this rough coverage of the genome (Table S1), only *YNL100W* and *RAS2,* which are located 3 kilobases (kb) apart on Chromosome 14, differed in allele frequencies between the two pools. In both genes, the S288c allele was the frequent allele in the “low” pool and the SK1 allele was the frequent allele in the “high” pool (Figure 2). The probability of finding at random two genes (out of 132) with such differences in allele frequencies between the two DNA pools is rather low even after the stringent Bonferroni correction for multiple tests (*p* < 0.014).

**Figure 3:**
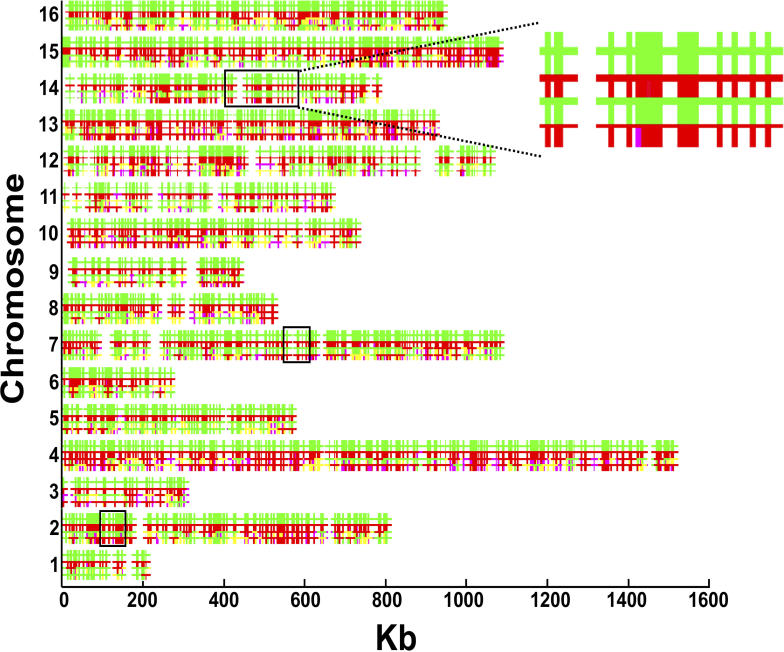
Hybridization of DNA from Parents and Pools of Segregants (“Low Tail” and “High Tail”) to Affymetrix S98 Microarrays For each chromosome, the top horizontal line (green) represents hybridizations of S288c DNA and the second line (red) represents hybridizations of SK1 DNA. The third and the fourth horizontal lines represent the hybridizations of the “low” and the “high” pools, respectively. Each horizontal array (comprised of four lines) represents a given yeast chromosome and the physical genomic positions along the chromosome. The small vertical bars represent probes containing polymorphisms between strains SK1 and S288c (alleles are colored according to their parental colors). The small vertical bars on the third and fourth lines of each chromosome represent the inherited allele in the pools: green is S288c and red is SK1. Inheritance of a mixture of alleles is marked either yellow (composition closer to S288c) or pink (closer to SK1). Three regions show consistent inherited differences in allele frequencies between the low and the high pools (boxes). These regions are located on Chromosome 2 (95–157 kb from the left end), Chromosome 7 (500–612 kb), and Chromosome 14 (400–585 kb).

A genome-wide discovery of SNPs, based on hybridization of genomic DNA to Affymetrix S98 yeast microarrays [25] was carried out using these two DNA pools. We compared probe intensities between five SK1 DNA hybridizations and five S288c hybridizations. As a result, we identified ~4,000 probes containing polymorphisms between these parental strains. Both the “high” and the “low” DNA pools were then hybridized to microarrays to identify differences in frequency of SNP's alleles. We detected three candidate regions, on Chromosomes 2, 7, and 14, each larger than 50 kb (with at least 20 differentiating SNPs). The SK1 allele was highly frequent in the “high” pool and the S288c allele in the “low” pool (Figure 3). The region detected on Chromosome 14 contained the genes *RAS2* and *YNL100W,* also detected by sequencing of the DNA pools (Figure 2).

Allele frequencies were verified by individual sequencing of the gene *RAS2* in 12 progeny from each pool. All 12 individuals from the “high” pool had the SK1 allele, whereas 11 out of 12 individuals from the “low” pool had the S288c allele. Based on further sequencing of the two DNA pools at 25-kb intervals flanking *RAS2* on both sides of the gene, the boundaries of this “candidate region” were determined to be 75 kb downstream and 100 kb upstream to *RAS2* (Figures 2 and S1). Following its identification by the genome-wide screen and by the individual SNP approach, this “candidate region” on Chromosome 14, which contains about 100 genes, was further studied in more detail.

Based on gene annotation and expression profile during meiosis [23], we chose 12 genes in this region that might be involved in sporulation/meiosis (Table S2). For each of the 12 candidates, we examined the possible contribution to the sporulation difference between the strains SK1 and S288c, by single reciprocal hemizygosity analysis [4]. The analysis is based on a phenotypic comparison between two hybrid strains, hemizygotic for either the SK1 or S288c allele in the gene of interest, but otherwise identical. Hemizygosity was achieved by deletion of the “other” allele (see Materials and Methods).

**Figure 4:**
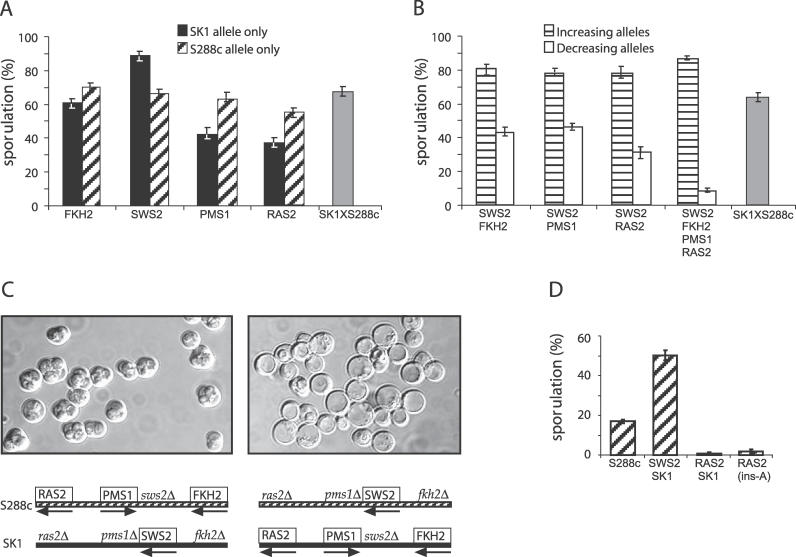
Effects of Reciprocal Hemizygosity and of Allele Replacements on Sporulation (A) Sporulation efficiency of pairs of hybrid strains (S288c × SK1) with single-gene heterozygous deletions. Strains deleted for the S288c allele (only the SK1 allele is present) are presented by black bars and the isogenic strains deleted for the corresponding SK1 allele (only the S288c allele is present) are presented as diagonally hatched bars. The non-deleted hybrid is presented for reference (gray bar). (B) Sporulation efficiency of double-gene and four-gene deletion mutants. Every pair consisted of two isogenic hybrid strains (S288c × SK1), each with two (or four) hemizygosities: One strain had deletions of the two (or four) sporulation-promoting alleles (empty bars) and the other had deletions of the corresponding sporulation-inhibiting alleles (bars with horizontal lines). (C) Sporulation of the four-gene deletion mutants. In the hybrid strain containing the four sporulation-promoting alleles (left microscopic image), almost all cells formed asci, whereas in the strain with the sporulation-inhibiting alleles (right image), most of the cells did not form asci. The genotypes of the two “reciprocal” strains are given below each image. (D) Sporulation efficiencies of a diploid S288c strain and two isogenic allele-replacement strains, one containing the two *SWS2* alleles from strain SK1 and the other containing the two *RAS2* alleles from SK1. A fourth isogenic strain contains, homozygotically, only a single additional A in the promoter poly-A stretch of *RAS2,* as found in strain SK1. For each strain, sporulation was assessed four times. The average sporulation efficiencies and their confidence intervals (*p* = 0.95) are shown.

Unlike the assessment of sporulation phenotypes of the original segregants, for all reciprocal-hemizygosity and allele-replacement tests we determined sporulation efficiency after 48 h in liquid sporulation medium. Significant differences in sporulation efficiencies (10%–20%) were detected between reciprocal hemizygosity strains of four genes: *FKH2, PMS1, RAS2,* and *SWS2* (Figure 4A). Counterintuitive to the phenotype of strain S288c (low sporulation efficiency), the presence of the sole S288c allele for each of the genes *FKH2, PMS1,* or *RAS2* resulted in higher sporulation efficiency compared to the presence of the single SK1 allele (in the same hybrid background). Only for the gene *SWS2,* the SK1 allele resulted in higher sporulation. These four genes were further analyzed as follows.

### Sequencing the Entire Open Reading Frame and Promoter Region

Non-synonymous SNPs that distinguish SK1 from S288c were detected in *FKH2* and *PMS1,* whereas in the gene *SWS2,* we found a synonymous SNP and SNPs in the promoter region. Two SNPs were found in the promoter region of *RAS2* (Table S3).

### Levels of RNA and Protein

Since neither *RAS2* nor *SWS2* contained non-synonymous SNPs, we assessed the expression pattern of their two alleles. RNA levels were assessed by RT-PCR in the hemizygous, hybrid genetic background at various time points during sporulation. The levels of RNA were compared between two hybrid strains (SK1 × S288c), which were hemizygotic to either *RAS2* or *SWS2.* Namely, one strain had only one copy of the SK1 allele of *SWS2* or *RAS2* (and none of the S288c allele) whereas the other hybrid strain had only one copy of the S288c allele of the same gene. The expression of the two different *SWS2* alleles at the beginning of sporulation differed significantly (RNA ratio for the alleles S288c/SK1 was 2.5; *p* < 0.015), whereas differences at later stages were less significant (Figure S2). RNA levels of the two *RAS2* alleles were quite similar throughout sporulation.

The protein levels of Ras2 and Sws2 for the SK1 and S288c alleles in the hemizygotic hybrid genetic backgrounds were assessed by Western blots. The protein levels of the products of the two *SWS2* alleles were significantly different from each other during sporulation (higher for the S288c allele; Figure S2). The two *RAS2* alleles did not differ in the amount of protein produced during sporulation (unpublished data).

### Double Hemizygotes

We generated double reciprocal hemizygotes for pairs of the four alleged genes, *SWS2* and *FKH2, SWS2* and *PMS1,* and *SWS2* and *RAS2.* Differences of ~40% in sporulation efficiencies were found between reciprocal strains (Figure 4B). The three strains containing the two sporulation-promoting alleles sporulated at 81%–84% efficiency, compared to 33%–48% of the strains containing the two sporulation-inhibiting alleles (Figure 4B).

### Assessment of Two Reciprocal Strains, Differing in All Four Genes

We compared sporulation efficiency of two strains, one containing all four sporulation-promoting alleles and the other containing all the sporulation-inhibiting ones. The hybrid strain containing the four sporulation-promoting alleles sporulated at a level of 86.5%, and the reciprocal strain, with the four sporulation-inhibiting alleles, sporulated at an efficiency of 9.1% (Figure 4B and 4C).

### Allele Replacements in *RAS2* and *SWS2*

A replacement in strain S288c (diploid) of the two S288c alleles of the gene *SWS2* by the corresponding SK1 alleles resulted in sporulation efficiency of 50.1% compared with 17.1% of the original, isogenic strain (Figure 4D). A similar replacement of the two copies of *RAS2* in strain S288c with two copies of the SK1 allele (including two SNPs in the promoter region) resulted in sporulation efficiency of 0.7% (Figure 4D). This result could explain the occurrence of segregants with sporulation efficiencies that are more extreme than their parents (Figure 1).

### Identification of the Causative SNP in the Gene *RAS2*

We applied direct mutagenesis to identify the causal SNP in *RAS2.* We thus added one adenine to the poly-A stretch in the promoter of the gene *RAS2* and generated an S288c diploid strain homozygous for this addition. This manipulation resulted in sporulation efficiency of 1.8%, compared with 17.1% of the original, isogenic S288c diploid strain (Figure 4D).

### Comparative Sequence Analysis

**Figure 5:**
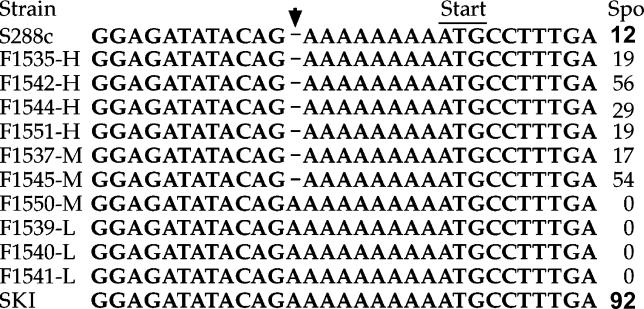
Sequence Comparisons of Part of the *RAS2* Promoter in Ten Winery Strains The published [27] assessment of sporulation efficiency are: H, high; M, moderate; L, low. Our assessment of sporulation efficiency (percent) is under “Spo.” The first codon of the open reading frame (ATG) is marked by “Start.” The black arrowhead indicates the deletion of adenine in the poly-A stretch. Based on their DNA sequences, the ten winery strains are closely related to each other and to strain S288c, whereas they differ from SK1 in many SNPs throughout the genome, by approximately 1 in 150 bp [26].

In a previous study [26], we compared various S. cerevisiae strains, including ten strains from a winery in California [27]. These strains were found to be genetically similar, although different in sporulation efficiency. We sequenced the four candidate genes in these ten winery strains. The four winery strains carrying the SK1 allele of *RAS2* in the promoter poly-A stretch did not sporulate at all, whereas the six strains having the S288c allele of *RAS2* sporulated at efficiencies of 15%–55% (Figure 5). All the ten winery strains carried the S288c alleles at all four genes except the A variation in the poly-A stretch of *RAS2.* However, since the four strains that carry the SK1 allele did not sporulate at all, it is possible that *RAS2* in these strains is linked to another dominant locus that blocks spore production.

## Discussion

The two budding yeast strains S288c and SK1 show a remarkable difference in sporulation efficiency, namely the rate at which they go through meiosis, a trait that might be of high evolutionary significance. A distribution of phenotypes observed in an F_2_ progeny of the cross between these strains suggests that this trait is genetically controlled in a quantitative fashion (Figure 1 and [14]). Deutschbauer and Davis [14] have recently mapped three genes controlling sporulation efficiency differences between S288c and SK1. They used segregants of backcross and DNA hybridizations to Affymetrix chips to do a linkage analysis that identified candidate regions on Chromosomes 7, 9, and 14. By reciprocal hemizygosity analysis of the genes in these three regions, they identified five genes with differences in sporulation between reciprocal hemizygous strains. These genes were further analyzed by allele replacement and site-directed mutagenesis. The genes *RME1, TAO3,* and *MKT1* are located on Chromosomes 7, 9, and 14, respectively, and were found to contribute to sporulation efficiency differences between the strains S288c and SK1.

The approach we took to identify sporulation QTLs combines a genome scan to identify inheritance bias in SNP alleles between phenotypically different DNA pools with reciprocal hemizygosity analysis [4] of individual candidate genes in the QTL region. DNA pools of 21 F_2_ segregants from each of the two tails of the sporulation efficiency distribution were sequenced at SNPs in 132 genes distributed over the genome. This step resulted in a definition of a “candidate region” on Chromosome 14, which was found to be strongly associated with differences in sporulation efficiency. In addition, this “candidate region” was confirmed in a genome-wide screen based on hybridizations of the DNA pools to Affymetrix oligonucleotide microarrays. The hybridization of pools yielded two additional regions that we did not follow up on, of which one on Chromosome 7 overlaps the *RME1* gene that Deutschbauer and Davis [14] identified. Thus, the use of DNA pools reduced costs significantly and was useful to the preliminary identification of QTL regions. However, using DNA pools, we did not identify the region on Chromosome 9 that was detected by Deutschbauer and Davis [14].

We have further analyzed 12 candidate genes in the Chromosome 14 QTL region by reciprocal hemizygosity analysis [4], and found four in which S288c alleles contributed differently to sporulation efficiency than SK1 alleles. Interestingly, the four genes that we identified were not included among the previously identified sporulation QTLs [14]. On the other hand, we have missed the gene *MKT1* in the Chromosome 14 QTL region [14], since we had not considered *MKT1* a candidate gene affecting sporulation or meiosis (this reflected the annotation of *MKT1* in the *Saccharomyces* Genome Database, http://www.yeastgenome.org). Combining the results of the two studies, the cluster on Chromosome 14 that controls sporulation contains five linked genes in a region of less than 60 kb. Strain SK1 has alleles with strong sporulation-promoting effects in the genes *SWS2* and *MKT1* and sporulation-reducing alleles in the genes *FKH2, PMS1,* and *RAS2,* whereas strain S288c has alleles of opposite effects in these five genes.

High genetic complexity of QTLs in the form of genomic clustering of genes that contribute to the same phenotype was reported previously [4,28–31], and here we identified four such genes that confer both promoting and inhibiting effects on the trait. Counterintuitively, however, the presence of the S288c alleles of the genes *FKH2, PMS1,* or *RAS2* resulted in higher sporulation efficiency compared to the presence of the corresponding SK1 alleles. In the gene *SWS2,* the SK1 allele resulted in higher sporulation efficiency than the S288c allele. The gene *SWS2* has a major contribution to the difference between the two parental strains, as demonstrated by the efficient sporulation (~50%, determined after 48 h in liquid sporulation medium) of the S288c strain in which the two *SWS2* alleles were replaced by the corresponding alleles from the strain SK1 (Figure 4D)*. MKT1,* the other gene with SK1 promoting allele in this region seems to have a milder effect on sporulation efficiency (about 15% sporulation, determined also after 48 h in liquid sporulation medium [14]). The net effect of these five linked genes, perhaps together with additional, yet undiscovered linked genes, is that this region confers high sporulation efficiency on the progeny that inherit it from parental strain SK1 (see hybridization of the “tail pools” in Figure 2).

To make the genetic architecture even more complex, *SWS2* and *PMS1* are two adjacent genes coded on opposite strands and thus share a short, common 3′ region. Could the effects of the adjacent genes be due to a single difference between the parental strains? Sporulation efficiency differences between reciprocal hemizygous strains increased from 20% when each gene was singly deleted, to 40% in the double-deletion reciprocal hemizygosity test (Figure 4). These results, together with their contribution in trans (i.e., strain SK1 has the sporulation-promoting allele in the gene *SWS2* and the sporulation-inhibiting allele in *PMS1*), suggest that *SWS2* and *PMS1* have distinct effects on the phenotype. It should be noted that in a recent study [32], *PMS1-MLH1* combinations from S288c and SK1 were found to cause defects in mismatch repair, with the causative SNP in *PMS1* being the SK1 allele R818K (G2453A; Table S3). The same mutation might be the sporulation-inhibiting SNP allele of SK1 in *PMS1*.

We also noticed a difference in the shape of the sporulation efficiency distribution among progeny of the cross SK1 × S288c between the study of Deutschbauer and Davis [14] and ours. Whereas we obtained a predominance of progeny with relatively high sporulation, like SK1 (Figure 1), they report more progeny that resembles S288c. A possible explanation for this difference is that in our study sporulation frequency was measured after 7 d on sporulation plates, whereas their data were obtained after 24 h in liquid sporulation medium. In any case, this difference in the phenotypic distribution suggests that the genes identified by both studies may contribute at different stages and in different ways to the kinetics of the sporulation process.

It appears that in the hybrid background, the S288c alleles of all four genes are dominant over the SK1 alleles with respect to sporulation, as each hemizygous strain, when deleted for the SK1 allele, showed a phenotype similar to the non-deleted hybrid (Figure 4A). In addition, all hemizygous strains having only the SK1 allele of *SWS2* (single-, double-, and four-gene mutants) sporulated at similar high efficiencies, higher than the non-deleted hybrid (Figure 4A and 4B). This suggests that at least in the hybrid background, *SWS2* masks the effects of the other three genes and that the S288c allele of *SWS2* inhibits sporulation. To test this phenomenon more thoroughly, sporulation efficiency of isogenic S288c strain with SK1 alleles of two, three, and all four genes should be determined. The differences in sporulation efficiencies between the two alleles of *SWS2,* as well as between the alleles of *RAS2,* however, are similar in both the heterozygous hybrid genetic background (reciprocal hemizygosities, Figure 4A) and the S288c homozygous background (gene replacements, Figure 4D).

In the case of *RAS2,* where the SK1 allele had an inhibiting effect on sporulation efficiency, our results suggest that an insertion of one additional adenine to a stretch of nine others in the promoter region decreases sporulation efficiency. The mechanism by which this change exerts its effect is not clear since the expression levels of both the RNA and protein did not differ between the reciprocal hemizygous strains throughout the sporulation process.

Other QTL studies failed to find marker-trait association in wild strains [4,14]. In the present study, the deletion of adenine in the poly-A stretch in the promoter region of *RAS2* distinguished the sporulation-proficient from the non-sporulating winery strains (Figure 5A). It is reasonable to hypothesize that in this case the genetic similarity between these wild winery strains [26] allowed the association between a DNA polymorphism and the phenotype. However, it is likely that this type of association in QTLs will be harder to find as genetic similarity drops. That the sporulation-proficient genome of strain SK1 carries the “low” sporulation alleles of *RAS2, PMS1,* and *FKH2* further emphasizes the caution required in using association-based methods to identify phenotypically relevant genetic variation.

Our results demonstrate the complexity of QTLs and have implications on similar studies in other organisms. Tight linkage between genes with alleles of opposite effects could result in difficulties to identify QTLs by linkage analysis where the net effect of alleles is low. Linked alleles of opposite effects can also reduce or “mask” variation estimates. One way to overcome such difficulties is to manipulate each gene in a suspected “candidate region” individually, and examine the phenotypic effects of such manipulations. This is more amenable in yeast, as was done here by analysis of reciprocal hemizygosity and allele replacements, and thus stresses the importance of this organism as a model for studying the genetics of complex traits.

## Materials and Methods

### Yeast strains.

The diploid strain SK1 was generated by mating two haploid strains having the SK1 background (NKY561 *[MATα; ho::hisG; ura3::hisG; lys2; leu2::hisG; trp1::hisG]* and NKY1059 *[MATa; ho::hisG; ura3::hisG; lys2; leu2::hisG; ade2; his4],* obtained from N. Kleckner's lab). The diploid S288c was generated by mating two haploids having the S288c genetic background (FY1338 *[MATα; ho; ura3*Δ*0; lys2*Δ*0; leu2*Δ*0; his3*Δ*200; met15*Δ*0; trp1*Δ*63]* and FY1344 *[MATa; ho; ura3*Δ*0; lys2*Δ*0; leu2*Δ*0; his3*Δ*200; met15*Δ*0; trp*Δ*63],* obtained from G. R. Fink's lab). Two S288c × SK1 hybrids, namely FY1344 × NKY561 and FY1338 × NKY1059, were sporulated to produce segregants, which were diploidized as described below.

The hybrids for the reciprocal hemizygosity analyses [4] resulted from mating between haploids of S288c background from the EUROSCARF deletion strains collection (BY4741) with, or without the *kanMX4*-cassette deletions (http://web.uni-frankfurt.de/fb15/mikro/euroscarf/complete.html) and haploid SK1 background (NE30 *[MATα; ho::hisG; leu2; trp1; ura3; lys2; ade2]*) with or without the *kanMX4* cassette deletions.

### Generation of diploid segregants and assessment of sporulation efficiency.

Assessment of sporulation efficiencies required the generation of diploid segregants. Thus, pYeS-HO, a 2-μm plasmid, containing *GAL1* promoter-*HO* and the *URA3* selection marker, was introduced into the hybrids S288c × SK1. Spores containing the plasmid were plated and grown on galactose containing medium for 24 h in order to switch their mating type, followed by growth on rich medium (to lose the plasmid). We then selected for colonies that did not contain the plasmid. Diploids were obtained by mating between haploid cells derived from the same segregant (with different mating types). We thus recovered 326 homozygous diploid segregants. Sporulation efficiency of the parents, the hybrids, and each of the segregants was assessed by counting (under the microscope) the number of asci produced by 200 cells after incubation of 1 wk on sporulation agar medium, at 30 °C.

### Media.

Sporulation agar plates contained 0.25% yeast extract, 1.5% potassium acetate, 0.05% glucose, and 1.5% agar. Sporulation efficiency of reciprocal hemizygous strains, as well as of strains with allele replacements, was assessed after overnight growth in standard rich medium—liquid YEPD (2% glucose, 2% bacto-peptone, 1% yeast extract, and 0.004% tryptophan and leucine) followed by 18 h in liquid YEPA (1% potassium acetate, 2% bacto-peptone, and 1% yeast extract) and 2 d in liquid SPM (0.3% potassium acetate and 0.02% raffinose).

### Selection of genes for sequencing (SNP discovery).

We have chosen for sequencing a total of 145 candidate genes in strain SK1 (Table S1). These genes were known to be associated with sporulation and were chosen in connection with another study. Some genes have been selected based on expression modules [33] and some on previous knowledge of their function (http://www.yeastgenome.org). In addition, some genes were chosen on the basis of two genome-wide expression studies during sporulation [23,24].

### Analysis of DNA hybridization to microarrays.

The identification of single feature polymorphisms (SFPs) between S288c and SK1 was done by hybridization of genomic DNA to Affymetrix S98 yeast microarrays [25]. The segregants' inheritance was similarly determined by using DNA pools of segregants from the two “tails” of the phenotypic distribution (Figure 1). For each probe, statistically significant biased inheritance [25] is presented by red or green marks (Figure 3); mixtures of the two alleles are presented as yellow or pink marks. To identify candidate regions, we looked for regions containing biased inheritance for both DNA pools across a number of neighboring probes. We thus decided on candidate regions larger than 50 kb with at least 20 differentiating SNPs with the same direction of bias.

### Reciprocal hemizygosity analyses.

Single mutants: For each tested gene, a PCR product amplified by external primers on a DNA template of the BY4741 deletion strain [34] was transformed into the SK1 haploid strain and selected on G418 plates. Transformation was verified for each gene by PCR, based on internal and external primers. Two independent transformants were tested for each gene. Mating between the BY4741 deletion strain and haploid SK1 or between the SK1 deletion strain and BY4741 generated the two reciprocal hybrids. Double mutants: S288c*SWS2*Δ was mated with SK1*FKH2*Δ, SK1*PMS1*Δ, or SK1*RAS2*Δ; similarly, SK1*SWS2*Δ was mated with S288c*FKH2*Δ, S288c*PMS1*Δ, or S288c*RAS2*Δ. Quadruple mutants: To generate two reciprocal hybrid strains differing in their alleles in four genes, we deleted the genes *FKH2* and *PMS1* in the strains SK1*RAS2*Δ and S288c*RAS2*Δ by homologous-recombination replacements of the ORFs with *URA3* and *LEU2,* respectively, and mated these strains with S288c*SWS2*Δ and SK1*SWS2*Δ, respectively.

### Determination of RNA and protein levels.

Kinetics of RNA and protein expression were obtained for *RAS2* and *SWS2* after 0, 5, and 10 h in SPM liquid medium (following overnight growth in YEPA medium). To compare the expression patterns of SK1 and S288c alleles of both genes, we used the corresponding reciprocal hemizygosity strains, with only a single allele present (in SK1 × S288c hybrid genetic background). RNA extraction was carried out using the Rneasy Midi kit (Qiagen, http://www1.qiagen.com) and RT-PCR was done as described [35], using *RDN18–1* (coding for ribosomal RNA) as a reference gene.

Strains were tagged with C terminal *Myc* tag as described [36] and tagging was verified by PCR. Extraction of proteins from meiotic cultures was performed according to Knop et al. [36]. Western blotting of proteins was also performed as described [36], using the mouse anti-Myc 9E11 monoclonal antibody (Santa Cruz Biotechnology, http://www.scbt.com) as a primary antibody.

### Allele replacements.

Due to their high transformation efficiency, we used strains of S288c genetic background for replacements of *RAS2* or *SWS2* with the corresponding alleles that were PCR-amplified from an SK1 strain. This was done in two steps: First, we transformed the gene *URA3* (a PCR product) into the *KanMX4* cassettes in the S288c*RAS2*Δ or S288c*SWS2*Δ strains; *URA3* was inserted downstream to the *KanMX4* cassette, so that transformation resulted in cells capable of growing on medium containing G418 and lacking uracil. Then, to insert the SK1 allele of *RAS2* or *SWS2,* 15 μg PCR product of the gene from SK1 were co-transformed with 0.15 μg of YPH425, a 2-μm plasmid containing the *LEU2* selection marker. Each gene was amplified by using primers from ~300 bp upstream and downstream to *RAS2* or *SWS2* loci, and DNA of SK1 as template. Transformed cells were plated on medium lacking leucine. These plates were replicated onto 5-FOA plates, and then to plates lacking uracil and other plates containing G418. Colonies that did not grow on the two latter media were selected. To verify that the cells were carrying the SK1 alleles, the replaced alleles *(RAS2* or *SWS2)* were amplified by PCR and sequenced. Haploids with S288c background and *RAS2* or *SWS2* alleles of SK1 were diploidized by the pYeS-HO plasmid, as described above.

Replacement of the poly-A stretch in S288c, at position −1 to −9 upstream of the ORF of *RAS2,* with that of SK1 (addition of one A) was done as follows: *RAS2* was amplified using primers 5′-TACGAGAGAATTACGGATAAAAAAACCAAG-3′ and 5′-CGTCTTCTTCCTCGTCTTCG-3′ using DNA of SK1 as template. The PCR product (containing the additional adenine, but not the other promoter SNP) was used for transformation of an S288c *ras2*Δ::KanMx4::*URA3* strain (described above). Transformed cells were selected on 5-FOA plates and then replicated onto plates lacking uracil and plates containing G418. Colonies that could not grow on the last two were selected. The single nucleotide modification was verified by PCR amplification and sequencing. Haploid transformants were diploidized as described above.

## Supporting Information

Figure S1 Boundaries of the Candidate Region on Chromosome 14 The ratios between SK1 and S288c alleles in the two DNA pools were measured at various positions upstream and downstream to *RAS2.* The continuous line represents the ratio between the SK1 and S288c alleles in the “high” DNA pool, whereas the dashed line represents the ratio between the S288c and SK1 alleles in the “low” DNA pool. The horizontal line represents allele ratio significantly different from 1:1 (ratio of 2.5). The candidate region boundaries were determined to be 75 kb upstream (540 kb) and 100 kb downstream (365 kb) of the gene *RAS2.* The four sporulation-associated genes identified in the present study are marked with arrows.
(277 KB AI).

Figure S2 The Expression Patterns of the Two *SWS2* Alleles during Sporulation (A) Protein levels produced by the two *SWS2* alleles during sporulation are significantly different from each other (higher for the S288c allele).
(B) The RNA levels produced by the two *SWS2* alleles at the beginning of sporulation differ significantly (RNA ratio for the alleles S288c/SK1 is 2.5; *p* < 0.015), whereas differences at later stages are not significant.
(306 KB AI).

Table S1 Description of the Discovered SNPs (291 KB DOC)

Table S2 Description of 12 Genes Examined by Reciprocal Hemizygosity (81 KB DOC)

Table S3 Positions of SNPs in the Four Newly Identified Sporulation Genes (139 KB DOC)

### Accession Numbers

The UniProt (http://www.pir.uniprot.org) accession numbers for the genes discussed in this paper are *FKH2* (P41813), *MKT1* (P40850), *PMS1* (P14242), *RAS2* (P01120), *RME1* (P32338), *SPS18* (P32572), *SWS2* (P53937), *TAO3* (P40468), and *YNL100W* (P50945).

## Abbreviations

bp: base pair
kb: kilobase
QTL: quantitative trait locus
SNP: single nucleotide polymorphism
