# Supplementary material for: Four Linked Genes Participate in Controlling Sporulation Efficiency in Budding Yeast
Source: PLoS Genet. 2006 Nov 17;2(11):e195. doi: 10.1371/journal.pgen.0020195 (PMC1636695; doi:10.1371/journal.pgen.0020195)
Supplement: Supplementary file 1 [file pgen.0020195.st001.doc]

**Supplementary Table 1 -** Characterization of the discovered SNPs

|  | **Systematic name** | **Standard name** | **Chromosome** | **Promoter bp*** | **ORF bp**** | **No. of**  **SNPs in promoter** | **No. of**  **SNPs in ORF** | **No. of**  **SNPs changing AA***** |
| --- | --- | --- | --- | --- | --- | --- | --- | --- |
| 1 | *YBL084C* | *CDC27* | 2 | 296 | 100 | 2 | 0 | 0 |
| 2 | *YBR063C* |  | 2 | 326 | 269 | 2 | 2 | 2 |
| 3 | *YBR106W* | *CDC28* | 2 | 111 | 489 | 0 | 1 | 0 |
| 4 | *YBR112C* | *SSN6* | 2 | 275 | 0 | 1 | 0 | 0 |
| 5 | *YBR148W* | *YSW1* | 2 | 388 | 77 | 1 | 0 | 0 |
| 6 | YBR180W | *DTR1* | 2 | 284 | 296 | 8 | 1 | 1 |
| 7 | *YBR184W* |  | 2 | 341 | 199 | 2 | 1 | 0 |
| 8 | *YBRO73w* | *RDH54* | 2 | 419 | 0 | 4 | 0 | 0 |
| 9 | *YCL055W* | *KAR4* | 3 | 363 | 0 | 0 | 0 | 0 |
| 10 | *YCR084C* | *TUP1* | 3 | 505 | 0 | 2 | 0 | 0 |
| 11 | *YDL154W* | *MSH5* | 4 | 344 | 371 | 3 | 0 | 0 |
| 12 | *YDL155W* | *CLB3* | 4 | 230 | 280 | 3 | 0 | 0 |
| 13 | *YDL174C* | *DLD1* | 4 | 250 | 0 | 2 | 0 | 0 |
| 14 | *YDL194W* | *SNF3* | 4 | 114 | 386 | 0 | 2 | 1 |
| 15 | *YDL239C* | *ADY3* | 4 | 340 | 360 | 5 | 6 | 4 |
| 16 | *YDR037w* | *KRS1* | 4 | 0 | 290 | 0 | 27 | 1 |
| 17 | *YDR042C* |  | 4 | 306 | 334 | 3 | 2 | 1 |
| 18 | *YDR052C* | *DBF4* | 4 | 387 | 0 | 2 | 0 | 0 |
| 19 | *YDR113C* | *PDS1* | 4 | 380 | 350 | 2 | 1 | 1 |
| 20 | *YDR118W* | *APC4* | 4 | 565 | 70 | 8 | 1 | 0 |
| 21 | *YDR207C* | *UME6* | 4 | 0 | 589 | 0 | 6 | 0 |
| 22 | *YDR218C* | *SPR28* | 4 | 306 | 274 | 3 | 1 | 1 |
| 23 | *YDR253C* | *MET32* | 4 | 351 | 384 | 4 | 1 | 1 |
| 24 | *YDR273W* | *DON1* | 4 | 378 | 347 | 6 | 3 | 0 |
| 25 | *YDR285W* | *ZIP1* | 4 | 311 | 309 | 3 | 1 | 0 |
| 26 | *YDR310C* | *SUM1* | 4 | 390 | 0 | 3 | 0 | 0 |
| 27 | *YDR374C* |  | 4 | 528 | 32 | 10 | 0 | 0 |
| 28 | *YDR403W* | *DIT1* | 4 | 321 | 289 | 2 | 1 | 0 |
| 29 | *YDR446W* | *ECM11* | 4 | 342 | 358 | 9 | 4 | 3 |
| 30 | *YDR451C* | *YHP1* | 4 | 0 | 527 | 0 | 0 | 0 |
| 31 | *YDR522C* | *SPS2* | 4 | 301 | 339 | 2 | 1 | 1 |
| 32 | *YDR523C* | *SPS1* | 4 | 186 | 304 | 1 | 0 | 0 |
| 33 | *YEL023C* |  | 5 | 346 | 239 | 2 | 1 | 0 |
| 34 | *YER115C* | *SPR6* | 5 | 271 | 369 | 3 | 1 | 1 |
| 35 | *YER179W* | *DMC1* | 5 | 291 | 409 | 5 | 2 | 1 |
| 36 | *YFL033C* | *RIM15* | 6 | 442 | 0 | 8 | 0 | 0 |
| 37 | *YFR028C* | *CDC14* | 6 | 550 | 0 | 2 | 0 | 0 |
| 38 | *YFR034C* | *PHO4* | 6 | 132 | 458 | 1 | 0 | 0 |
| 39 | *YGL003C* | *HCT1* | 7 | 567 | 0 | 2 | 0 | 0 |
| 40 | *YGL033W* | *HOP2* | 7 | 341 | 279 | 8 | 2 | 2 |
| 41 | *YGL081W* |  | 7 | 232 | 358 | 1 | 4 | 0 |
| 42 | *YGL116W* | *CDC20* | 7 | 363 | 225 | 2 | 2 | 0 |
| 43 | *YGL137W* | *SEC27* | 7 | 302 | 298 | 0 | 1 | 0 |
| 44 | *YGL156W* | *AMS1* | 7 | 362 | 258 | 1 | 2 | 0 |
| 45 | *YGL170C* | *SPO74* | 7 | 318 | 369 | 2 | 2 | 2 |
| 46 | *YGL181W* | *GTS1* | 7 | 0 | 495 | 0 | 0 | 0 |
|  | **Systematic name** | **Standard name** | **Chromosome** | **Promoter bp*** | **ORF bp**** | **No. of**  **SNPs in promoter** | **No. of**  **SNPs in ORF** | **No. of**  **SNPs changing AA***** |
| 47 | *YGL183C* |  | 7 | 351 | 314 | 1 | 0 | 0 |
| 48 | *YGL192W* | *SPO8* | 7 | 823 | 327 | 9 | 2 | 1 |
| 49 | *YGL251C* | *HFM1* | 7 | 311 | 279 | 0 | 1 | 0 |
| 50 | *YGR044C* | *RME1* | 7 | 0 | 575 | 0 | 3 | 0 |
| 51 | *YGR059W* | *SPR3* | 7 | 343 | 337 | 6 | 0 | 0 |
| 52 | *YGR109C* | *CLB6* | 7 | 340 | 141 | 1 | 2 | 1 |
| 53 | *YGR273C* |  | 7 | 341 | 279 | 5 | 1 | 1 |
| 54 | *YHL022C* | *SPO11* | 8 | 349 | 229 | 9 | 0 | 0 |
| 55 | *YHL027W* | *RIM101* | 8 | 0 | 578 | 0 | 6 | 3 |
| 56 | *YHR014W* | *SPO13* | 8 | 312 | 368 | 1 | 1 | 1 |
| 57 | *YHR015W* | *MIP6* | 8 | 243 | 377 | 1 | 1 | 0 |
| 58 | *YHR124W* | *NDT80* | 8 | 265 | 290 | 0 | 1 | 0 |
| 59 | *YHR152W* | *SPO12* | 8 | 539 | 0 | 3 | 0 | 0 |
| 60 | *YHR153C* | *SPO16* | 8 | 341 | 304 | 4 | 4 | 4 |
| 61 | *YHR157W* | *REC104* | 8 | 304 | 316 | 2 | 2 | 1 |
| 62 | *YHR166C* | *CDC23* | 8 | 422 | 198 | 3 | 0 | 0 |
| 63 | *YHR184W* | *SPO3* | 8 | 0 | 190 | 0 | 0 | 0 |
| 64 | *YHRCdelta14* | | 8 | 0 | 420 | 0 | 1 | 0 |
| 65 | *YIL031W* | *SMT4* | 9 | 302 | 248 | 2 | 1 | 1 |
| 66 | *YIL072W* | *HOP1* | 9 | 333 | 367 | 13 | 2 | 1 |
| 67 | *YIL139C* | *REV7* | 9 | 292 | 78 | 2 | 0 | 0 |
| 68 | *YJL005W* | *CYR1* | 10 | 26 | 400 | 0 | 1 | 0 |
| 69 | *YJL013C* | *MAD3* | 10 | 144 | 370 | 0 | 0 | 0 |
| 70 | *YJL106W* | *IME2* | 10 | 813 | 197 | 2 | 0 | 0 |
| 71 | *YJL167W* | *ERG20* | 10 | 271 | 319 | 0 | 0 | 0 |
| 72 | *YJR036C* | *HUL4* | 10 | 334 | 386 | 0 | 4 | 4 |
| 73 | *YJR094C* | *IME1* | 10 | 0 | 607 | 0 | 0 | 0 |
| 74 | *YKL042W* | *SPC42* | 11 | 158 | 0 | 1 | 0 | 0 |
| 75 | *YKL062W* | *MSN4* | 11 | 490 | 0 | 2 | 0 | 0 |
| 76 | *YKL112W* | *ABF1* | 11 | 520 | 0 | 0 | 0 | 0 |
| 77 | *YKR005C* |  | 11 | 371 | 279 | 1 | 1 | 1 |
| 78 | *YKR016W* |  | 11 | 164 | 346 | 4 | 1 | 1 |
| 79 | *YKR031C* | *SPO14* | 11 | 567 | 46 | 5 | 0 | 0 |
| 80 | *YLL046C* | *RNP1* | 12 | 261 | 219 | 2 | 2 | 1 |
| 81 | *YLR013W* | *GAT3* | 12 | 0 | 564 | 0 | 1 | 0 |
| 82 | *YLR071C* | *RGR1* | 12 | 535 | 20 | 5 | 0 | 0 |
| 83 | *YLR096W* | *KIN2* | 12 | 329 | 266 | 1 | 0 | 0 |
| 84 | *YLR165c* | *PUS5* | 12 | 0 | 390 | 0 | 1 | 1 |
| 85 | *YLR182W* | *SWI6* | 12 | 0 | 573 | 0 | 0 | 0 |
| 86 | *YLR210W* | *CLB4* | 12 | 560 | 0 | 1 | 0 | 0 |
| 87 | *YLR213C* | *CRR1* | 12 | 200 | 0 | 1 | 0 | 0 |
| 88 | *YLR227C* | *ADY4* | 12 | 321 | 279 | 4 | 0 | 0 |
| 89 | *YLR263W* | *RED1* | 12 | 149 | 301 | 2 | 1 | 1 |
| 90 | *YLR308W* | *CDA2* | 12 | 338 | 297 | 6 | 1 | 1 |
| 91 | *YLR310C* | *CDC25* | 12 | 222 | 403 | 4 | 1 | 0 |
| 92 | *YLR329W* | *REC102* | 12 | 418 | 0 | 9 | 0 | 0 |
| 93 | *YLR343W* | *GAS2* | 12 | 352 | 238 | 4 | 0 | 0 |
|  | **Systematic name** | **Standard name** | **Chromosome** | **Promoter bp*** | **ORF bp**** | **No. of**  **SNPs in promoter** | **No. of**  **SNPs in ORF** | **No. of**  **SNPs changing AA***** |
| 94 | *YLR394W* | *CST9* | 12 | 304 | 316 | 0 | 0 | 0 |
| 95 | *YLR399C* | *BDF1* | 12 | 0 | 593 | 0 | 0 | 0 |
| 96 | *YMR001C* | *CDC5* | 13 | 609 | 0 | 3 | 0 | 0 |
| 97 | *YMR021C* | *MAC1* | 13 | 566 | 64 | 4 | 1 | 0 |
| 98 | *YMR037C* | *MSN2* | 13 | 315 | 0 | 1 | 0 | 0 |
| 99 | *YMR063W* | *RIM9* | 13 | 505 | 46 | 7 | 0 | 0 |
| 100 | *YMR125W* | *STO1* | 13 | 480 | 58 | 3 | 0 | 0 |
| 101 | *YMR133W* | *REC114* | 13 | 318 | 367 | 0 | 1 | 0 |
| 102 | *YMR139W* | *RIM11* | 13 | 266 | 294 | 1 | 1 | 1 |
| 103 | *YMR148W* |  | 13 | 351 | 379 | 3 | 2 | 0 |
| 104 | *YMR154C* | *RIM13* | 13 | 463 | 93 | 8 | 0 | 0 |
| 105 | YMR199W | *CLN1* | 13 | 351 | 249 | 3 | 0 | 0 |
| 106 | *YMR306W* | *FKS3* | 13 | 298 | 367 | 0 | 0 | 0 |
| 107 | *YNL012W* | *SPO1* | 14 | 336 | 214 | 1 | 0 | 0 |
| 108 | *YNL033W* |  | 14 | 305 | 290 | 4 | 0 | 0 |
| 109 | *YNL098C* | *RAS2* | 14 | 323 | 188 | 2 | 0 | 0 |
| 110 | *YNL100W* |  | 14 | 352 | 278 | 2 | 0 | 0 |
| 111 | *YNL155W* |  | 14 | 351 | 359 | 5 | 1 | 1 |
| 112 | *YNL196C* | *SLZ1* | 14 | 346 | 174 | 2 | 2 | 1 |
| 113 | *YNL204C* | *SPS18* | 14 | 0 | 506 | 0 | 5 | 3 |
| 114 | *YNL236W* | *SIN4* | 14 | 260 | 280 | 2 | 2 | 1 |
| 115 | *YNL307C* | *MCK1* | 14 | 368 | 219 | 4 | 1 | 0 |
| 116 | *YNL330C* | *RPD3* | 14 | 118 | 432 | 1 | 1 | 1 |
| 117 | *YNR034W* | *SOL1* | 14 | 323 | 197 | 3 | 0 | 0 |
| 118 | *YOL004W* | *SIN3* | 15 | 595 | 0 | 4 | 0 | 0 |
| 119 | *YOL024W* |  | 15 | 307 | 282 | 0 | 0 | 0 |
| 120 | *YOL067C* | *RTG1* | 15 | 179 | 26 | 1 | 0 | 0 |
| 121 | *YOL068C* | *HST1* | 15 | 123 | 381 | 0 | 0 | 0 |
| 122 | *YOL091W* | *SPO21* | 15 | 332 | 333 | 2 | 3 | 1 |
| 123 | *YOL132W* |  | 15 | 252 | 298 | 0 | 1 | 0 |
| 124 | *YOR032C* | *HMS1* | 15 | 570 | 0 | 5 | 0 | 0 |
| 125 | *YOR074C* | *CDC21* | 15 | 477 | 115 | 1 | 0 | 0 |
| 126 | *YOR177C* | *MPC54* | 15 | 140 | 365 | 1 | 2 | 1 |
| 127 | *YOR190W* | *SPR1* | 15 | 221 | 349 | 2 | 1 | 1 |
| 128 | *YOR209C* | *NPT1* | 15 | 281 | 249 | 1 | 0 | 0 |
| 129 | *YOR229W* | *WTM2* | 15 | 0 | 568 | 0 | 4 | 0 |
| 130 | *YOR242C* | *SSP2* | 15 | 218 | 352 | 3 | 4 | 3 |
| 131 | *YOR249C* | *APC5* | 15 | 231 | 349 | 0 | 4 | 1 |
| 132 | *YOR313C* | *SPS4* | 15 | 343 | 237 | 3 | 1 | 1 |
| 133 | *YOR339C* | *UBC11* | 15 | 226 | 349 | 2 | 2 | 0 |
| 134 | *YOR351C* | *MEK1* | 15 | 361 | 314 | 1 | 2 | 1 |
| 135 | *YOR365C* |  | 15 | 234 | 326 | 4 | 2 | 0 |
| 136 | *YOR391C* |  | 15 | 548 | 29 | 9 | 1 | 0 |
| 137 | *YPL021W* | *ECM23* | 16 | 311 | 249 | 2 | 1 | 1 |
| 138 | *YPL061W* | *ALD6* | 16 | 353 | 247 | 4 | 0 | 0 |
| 139 | *YPL118W* | *MRP51* | 16 | 322 | 268 | 4 | 0 | 0 |
| 140 | *YPL121C* | *MEI5* | 16 | 354 | 356 | 4 | 1 | 0 |
|  | **Systematic name** | **Standard name** | **Chromosome** | **Promoter bp*** | **ORF bp**** | **No. of**  **SNPs in promoter** | **No. of**  **SNPs in ORF** | **No. of**  **SNPs changing AA***** |
| 141 | *YPR007C* | *SPO69/REC8* | 16 | 353 | 267 | 6 | 3 | 0 |
| 142 | *YPR026W* | *ATH1* | 16 | 230 | 0 | 3 | 0 | 0 |
| 143 | *YPR077C* |  | 16 | 411 | 249 | 3 | 0 | 0 |
| 144 | *YPR078C* |  | 16 | 460 | 0 | 9 | 0 | 0 |
| 145 | *YPR120C* | *CLB5* | 16 | 615 | 0 | 5 | 0 | 0 |

* Length of the sequenced region upstream to the first ATG.

** Length of the sequenced region of the ORF.

*** Number of non-synonymous SNPs.
