# Supplementary material for: Four Linked Genes Participate in Controlling Sporulation Efficiency in Budding Yeast
Source: PLoS Genet. 2006 Nov 17;2(11):e195. doi: 10.1371/journal.pgen.0020195 (PMC1636695; doi:10.1371/journal.pgen.0020195)
Supplement: Supplementary file 2 [file pgen.0020195.st002.doc]

**Supplementary Table 2 -** Description of 12 genes examined by reciprocal hemizygosity.

| **Systematic Name** | **Standard Name** | **Annotation*** |
| --- | --- | --- |
| *YNL068C* | *FKH2* | Transcription factor of the forkhead family that regulates the cell cycle and pseudohyphal growth; also involved in chromatin silencing at HML and HMR; potential Cdc28p substrate |
| YNL069C | *RPL16B* | N-terminally acetylated protein component of the large (60S) ribosomal subunit, binds to 5.8 S rRNA; has similarity to Rpl16Ap, E. coli L13 and rat L13a ribosomal proteins; transcriptionally regulated by Rap1p |
| *YNL072W* | *RNH35* | Ribonuclease H2 catalytic subunit, removes RNA primers during Okazaki fragment synthesis; cooperates with Rad27p nuclease |
| *YNL073W* | *MSK1* | Mitochondrial lysine-tRNA synthetase, required for import of both aminoacylated and deacylated forms of tRNA(Lys) into mitochondria |
| *YNL081C* | *SWS2* | Putative mitochondrial ribosomal protein of the small subunit, has similarity to E. coli S13 ribosomal protein |
| *YNL082W* | *PMS1* | Required for mismatch repair in mitosis and meiosis, low levels of postmeiotic segregation, and high spore viability, dispensable for homeologous recombination; MutL homolog, similar to Mlh1p, associates with Mlh1p, possibly forming a heterodimer, Pms1p and Msh1p act in concert to bind to a Msh2p-heteroduplex complex containing a G-T mismatch |
| *YNL083W* | *SAL1* | Probable transporter, member of the Ca2+-binding subfamily of the mitochondrial carrier family, with two EF-hand motifs; Pet9p and Sal1p have an overlapping function critical for viability; polymorphic in different S. cerevisiae strains |
| *YNL093W* | *YPT53* | Involved in vacuolar protein sorting and endocytosis; GTP-binding protein of the rab family |
| *YNL098C* | *RAS2* | GTP-binding protein involved in growth on nonfermentable carbon sources, the starvation response, sporulation, pseudohyphal growth, and aging; homolog of Ras proto-oncogenes |
| *YNL100W* |  | Hypothetical ORF |
| *YNL104C* | *LEU4* | Alpha-isopropylmalate synthase (2-isopropylmalate synthase); the main isozyme responsible for the first step in the leucine biosynthesis pathway |
| *YNL117W* | *MLS1* | carbon-catabolite sensitive malate synthase |

*Annotation taken from *Saccharomyces* Genome Database (SGD).
