# Supplementary material for: Four Linked Genes Participate in Controlling Sporulation Efficiency in Budding Yeast
Source: PLoS Genet. 2006 Nov 17;2(11):e195. doi: 10.1371/journal.pgen.0020195 (PMC1636695; doi:10.1371/journal.pgen.0020195)
Supplement: Supplementary file 3 [file pgen.0020195.st003.doc]

**Supplementary Table 3 -** Positions of SNPs in the four genes identified as sporulation QTLs.

| **Sequence** | **Strain** | ***PMS1* – position and SNPs** | | | | | | | | | | | | | | | | | | | | | | | | | | | | | | | | |
| --- | --- | --- | --- | --- | --- | --- | --- | --- | --- | --- | --- | --- | --- | --- | --- | --- | --- | --- | --- | --- | --- | --- | --- | --- | --- | --- | --- | --- | --- | --- | --- | --- | --- | --- |
|  |  | -245 | -89 | -70 | -34 | -16 | -2 | 122 | 213 | 258 | 333 | 335 | 465 | 552 | 558 | 708 | 711 | 810 | 855 | 939 | 1150 | 1199 | 1246 - 1251 | | | | | 1538 | 1575 | 1782 | 1821 | 2322 | 2364 | 2453 |
| DNA | S288c | G | T | A | A | G | G | A | C | T | G | T | C | T | T | A | T | G | G | T | T | C | ACTACT | | | | | A | G | T | A | T | G | G |
|  | SK1 | C | C | G | G | A | C | G | T | C | C | C | T | A | C | G | C | C | A | A | G | G | ACTTGTGAAGGAACTACT | | | | | T | C | C | G | G | A | A |
| Protein* | S288c |  |  |  |  |  |  | **N** |  |  |  | **I** |  |  |  |  |  |  |  |  | **F** | T | TT | | | | | Y |  |  |  |  |  | R |
|  | SK1 |  |  |  |  |  |  | **S** |  |  |  | **T** |  |  |  |  |  |  |  |  | **V** | S | TCEGTT | | | | | F |  |  |  |  |  | K |
|  |  |  |  |  |  |  |  |  |  |  |  |  |  |  |  |  |  |  |  |  |  |  |  |  |  |  |  |  |  |  |  |  |  |  |
| **Sequence** | **Strain** | ***FKH2* – position and SNPs** | | | | | | | | | | | |  |  |  |  |  |  |  |  |  |  |  |  |  |  |  |  |  |  |  |  |  |
|  |  | -225 | 921 | 966 | 987 | 990 | 1191 | 1200 | 1498 | 1614 | 1720 | 1785 | 2372 |  |  |  |  |  |  |  |  |  |  |  |  |  |  |  |  |  |  |  |  |  |
| DNA | S288c | A | C | T | G | C | G | G | A | C | T | C | G |  |  |  |  |  |  |  |  |  |  |  |  |  |  |  |  |  |  |  |  |  |
|  | SK1 | G | T | C | C | T | A | A | C | G | C | G | A |  |  |  |  |  |  |  |  |  |  |  |  |  |  |  |  |  |  |  |  |  |
| Protein* | S288c |  |  |  |  |  |  |  | **K** |  | **S** |  | **S** |  |  |  |  |  |  |  |  |  |  |  |  |  |  |  |  |  |  |  |  |  |
|  | SK1 |  |  |  |  |  |  |  | **Q** |  | **P** |  | **N** |  |  |  |  |  |  |  |  |  |  |  |  |  |  |  |  |  |  |  |  |  |
|  |  |  |  |  |  |  |  |  |  |  |  |  |  |  |  |  |  |  |  |  |  |  |  |  |  |  |  |  |  |  |  |  |  |  |
| **Sequence** | **Strain** | ***SWS2* – position and SNPs** | | | | | | | | | | | | |  |  |  |  |  |  |  |  |  |  |  |  |  |  |  |  |  |  |  |  |
|  |  | -289 | -204 - -205 | | | | -191 | -112 | -97 - -91 | | | | -35 | 21 |  |  |  |  |  |  |  |  |  |  |  |  |  |  |  |  |  |  |  |  |
| DNA | S288c | A | AT | | | | A | T | AAAAAAA | | | | C | T |  |  |  |  |  |  |  |  |  |  |  |  |  |  |  |  |  |  |  |  |
|  | SK1 | G | AGTTCTCAACT | | | | G | C | AAAAAA | | | | G | G |  |  |  |  |  |  |  |  |  |  |  |  |  |  |  |  |  |  |  |  |
|  |  |  |  |  |  |  |  |  |  |  |  |  |  |  |  |  |  |  |  |  |  |  |  |  |  |  |  |  |  |  |  |  |  |  |
| **Sequence** | **Strain** | ***RAS2* – position and SNPs** | | | | | | | |  |  |  |  |  |  |  |  |  |  |  |  |  |  |  |  |  |  |  |  |  |  |  |  |  |
|  |  | -52 | -9 - -1 | | | | | | |  |  |  |  |  |  |  |  |  |  |  |  |  |  |  |  |  |  |  |  |  |  |  |  |  |
| DNA | S288c | A | AAAAAAAAA | | | | | | |  |  |  |  |  |  |  |  |  |  |  |  |  |  |  |  |  |  |  |  |  |  |  |  |  |
|  | SK1 | G | AAAAAAAAAA | | | | | | |  |  |  |  |  |  |  |  |  |  |  |  |  |  |  |  |  |  |  |  |  |  |  |  |  |

*In the protein sequence only non-synonymous SNPs are presented. SNPs resulting in amino-acids from different groups are in bold.
